# KMT2C/D COMPASS complex-associated diseases [K_CD_COM-ADs]: an emerging class of congenital regulopathies

**DOI:** 10.1186/s13148-019-0802-2

**Published:** 2020-01-10

**Authors:** William J. Lavery, Artem Barski, Susan Wiley, Elizabeth K. Schorry, Andrew W. Lindsley

**Affiliations:** 1 Division of Allergy and Immunology, Cincinnati Children’s Hospital Medical Center (CCHMC), 3333 Burnet Avenue, Cincinnati, OH 45229-3026 USA; 2 Division of Human Genetics, CCHMC, Cincinnati, OH USA; 3 Division of Developmental and Behavioral Pediatrics, CCHMC, Cincinnati, OH USA

**Keywords:** COMPASS complex, Epigenetics, Kabuki syndrome, Rubinstein-Taybi syndrome, Kleefstra syndrome, Lysine methyltransferase, Demethylase, Histone deacetylase, KMT2D, KMT2C, KDM6A, EP300, CBP, EHMT1

## Abstract

The type 2 lysine methyltransferases KMT2C and KMT2D are large, enzymatically active scaffold proteins that form the core of nuclear regulatory structures known as KMT2C/D COMPASS complexes (complex of proteins associating with Set1). These evolutionarily conserved proteins regulate DNA promoter and enhancer elements, modulating the activity of diverse cell types critical for embryonic morphogenesis, central nervous system development, and post-natal survival. KMT2C/D COMPASS complexes and their binding partners enhance active gene expression of specific loci via the targeted modification of histone-3 tail residues, in general promoting active euchromatic conformations. Over the last 20 years, mutations in five key COMPASS complex genes have been linked to three human congenital syndromes: Kabuki syndrome (type 1 [*KMT2D*] and 2 [*KDM6A*]), Rubinstein-Taybi syndrome (type 1 [*CBP*] and 2 [*EP300*]), and Kleefstra syndrome type 2 (*KMT2C*). Here, we review the composition and biochemical function of the KMT2 complexes. The specific cellular and embryonic roles of the KMT2C/D COMPASS complex are highlight with a focus on clinically relevant mechanisms sensitive to haploinsufficiency. The phenotypic similarities and differences between the members of this new family of disorders are outlined and emerging therapeutic strategies are detailed.

## Background

Breakthroughs in DNA sequencing technologies over the last decade have revolutionized the clinical diagnosis of genetic diseases and driven the development of an ever-expanding molecular biology toolbox [1]. Sequencing-dependent, high-throughput technologies, such as RNA sequencing (RNA-Seq) and chromatin immunoprecipitation sequencing (ChIP-Seq), have empowered genome-wide characterization of gene expression and chromatin regulation [2]. These techniques have also revealed that dysregulation of epigenetic mechanisms, including aberrant post-translational modification of histone, is associated with a wide array of human disease states, including cancer, immune dysfunction, and complex, multi-systemic congenital disease [3].

**Fig. 1:**
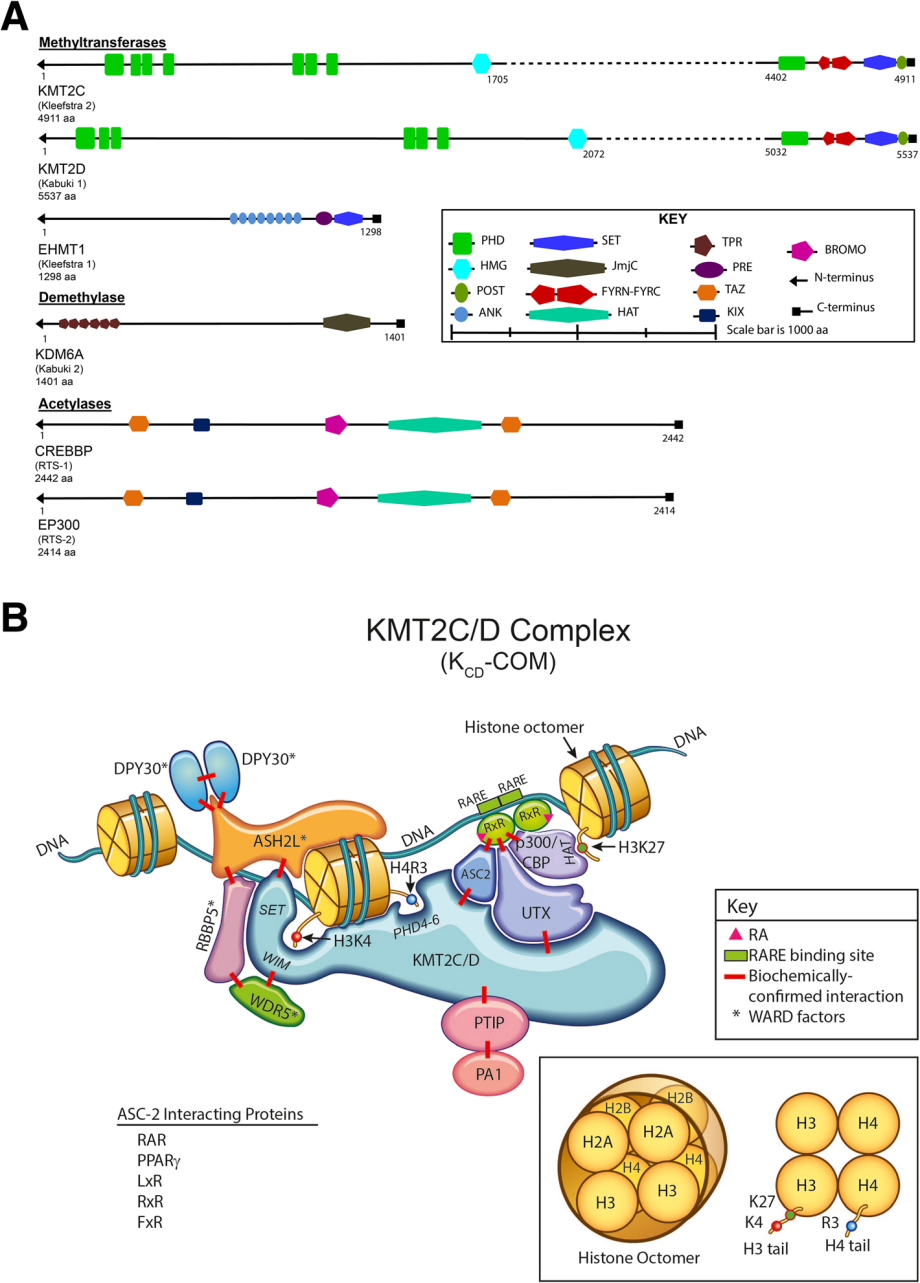
**a** Histone-modifying genes of interest (*KMT2C*, *KMT2D*, *EHMT1*, *KDM6A*, *CRBBP*, and *EP300*) functional domains scale schematic. Amino acids (aa), Plant homeodomain (PHD), high mobility group domain (HMG), F/Y-rich N-terminus domain (FYRN), F/Y-rich C-terminus domain (FYRC), SET domain (SET), post-SET domain (POST), pre-SET domain (PRE), Ankyrin repeat domain (ANK), JmjC domain (JmjC), tetratricopeptide repeat protein domain (TPR), transcriptional adapter zinc binding domain (TAZ), kinase-inducible domain interacting domain (KIX), bromodomain (BROMO), histone acetyltransferase domain (HAT). **b** Schematic of K_CD_COM. COMPASS-like complexes (K_CD_COMs, also historically known as ASC2-binding complexes, ASCOMs) bind multiple unique subunits (NCOA6/ASC2, KDM6A/UTX, PTIP, and PAGR1/PA1) and interact with chromatin via histone tail post-translational modifications and DNA binding cofactors. *RA* retinoic acid. WRAD WDR5 (WD repeat domain 5), RBBP5 (retinoblastoma binding protein 5), ASHL2 (absent, small or homeotic 2-like), and DPY-30 (Dumpy-30)

That developmentally important epigenetic mechanisms can go awry and can cause syndromic congenital disease is not surprising; however, the evidence establishing how such disease states are molecularly and mechanistically linked is only now beginning to emerge. The broader availability of clinical exome sequencing has uncovered significant phenotypic heterogeneity within diseases and highlighted overlap between congenital diseases previously thought of as being distinct. Though such incomplete penetrance, variable expressivity, and “phenocopying” phenomenon can confuse or delay a clinical diagnosis, these observations have also provided new mechanistic hints about how defects in seemingly diverse genes can contribute to similar phenotypic endpoints. Herein, we will discuss how defects in a developmentally important, promoter-activating and enhancer-commissioning pathways can link mutations in five different genes associated with three distinct congenital epigenetic diseases: Kabuki syndrome (type 1 [*KMT2D*] and 2 [*KDM6A*]), Rubinstein-Taybi syndrome (type 1 [*CBP*] and 2 [*EP300*]), and Kleefstra syndrome type 2 (*KMT2C*) (Fig. 1a). We will review the molecular components of the KMT2C/D COMPASS complex, its functional dependence on the histone acetylation enzymes p300 (encoded by *EP300*)/CBP, and the distinct and overlapping phenotypes linked with each of the three associated diseases. We will close with a discussion of potential strategies to target and mitigate promoter/enhancer dysfunction postnatally in this new class of “congenital histone-3 regulopathies.”

## KMT2C/D COMPASS complexes (K_CD_COMs)

### KMT2 core proteins

Type 2 lysine methyltransferases (KMT2) are a family of six mammalian, histone-modifying proteins (KMT2A-D, F and G) that catalyze mono-, di-, or trimethylation of the fourth lysine on the histone 3 tail (H3K4me1, H3K4me2, and H3K4me3, respectively) [4, 5]. Each individual KMT2 gene appears important for embryonic development, with murine knockout models of each of these six genes demonstrating embryonic/perinatal lethality [6]. KMT2 protein enzymatic activity is driven by the highly conserved SET domain, named for the three *Drosophila* proteins (Su(var)3-9, Enhancer-of-zeste, Trithorax) in which this ~ 130–amino acid peptide sequence was first identified [7, 8]. SET domain activity requires the methyl group donor co-factor S-adenosyl-l-methionine (SAM) and targets the ε amino group of the recipient lysine residue [9]. Targeting of specific lysine substrates (e.g., H3K4) by KMT2 proteins is not mediated by SET, as this domain is found in at least 51 other human proteins with a wide range of both histone (e.g., H3K9, H3K27, H3K36) and non-histone (e.g., DNMT3 RARA, p53) targets [5]. Substrate specificity appears to emerge from KMT2 proteins physically interacting with multiple protein cofactors, thus forming very large, multimeric regulatory complexes. Biochemical purification of the yeast protein Set1 from a large nuclear protein complex led to the first characterization of SET-domain protein cofactors. Termed the COMPASS complex (complex of proteins associating with Set1), homologous members of these protein cofactors were later identified in insect and mammalian cells and found to form similar nuclear complexes (COMPASS [KMT2F/G] and COMPASS-related/-like complexes [KMT2A-D]).

### COMPASS complex cofactors

Mammalian COMPASS and COMPASS-related complexes contain critical co-factors that enhance methyltransferase enzymatic activity and enforce substrate specificity. All KMT2 COMPASS complexes bind a critical, four-member subcomplex (called the WRAD or WARD complex) that interacts with the SET domain and enhances the methyltransferase catalytic activity by up to 600-fold [10, 11]. The WRAD complex contains the subunits WD repeat domain 5 (WDR5), retinoblastoma binding protein 5 (RBBP5), absent, small, or homeotic 2-like (ASHL2), and Dumpy-30 (DPY-30). The WRAD proteins can self-assemble independently of a KMT2 core protein, facilitate SET domain interaction with the nucleosome/H3 tail (Fig. 1b), and likely possess intrinsic methyltransferase activity independent of the SET domain-encoding KMT2 proteins they bind [12].

#### WRAD complex components

WRAD complex proteins are present at substantially higher cellular levels than the KMT2 core proteins and are linked with many non-KMT2 biochemical roles. These proteins interact via specific conserved motifs and domains as outlined below (also see Table 1(A) and Fig. 1b). The most highly expressed of these subunits is WDR5, a WD40 repeat—containing protein that contains two key interaction domains (Win, WDR5-interacting site; WBM, WDR5-binding motif). The WDR5 Win domain binds a conserved Win interaction motif (WIM) found on all KMT2 proteins [13, 14], whereas the WBM domain facilitates WDR5 binding to the RBBP5 core subunit (as well as other nuclear proteins and transcription factors) [15, 16]. The WDR5 Win domain also acts as a histone reader specific for the H3R2me2 marker associated with chromatin “poised” for transcription. The WRD5 interaction with H3R2, however, then blocks WRD5 binding to the KMT2 SET domain. Similarly, the WBM domain is promiscuous, allowing the WDR5 protein to interact with other chromatin-modifying complexes, such as the NSL and NuRD complexes. Like WDR5, RBBP5 also contains a WD40 motif and acts similarly as a scaffold protein, binding both WDR5 and the trithorax-like gene ASH2L. The RBBP5-ASH2L interaction is mediated via binding of a cluster of acidic residues (D/E box) on RBBP5 to the SPRY (spla and the ryanodine receptor) domain on ASH2L [17]. ASH2L, homologous to the Drosophila gene Ash2, contains a histone-binding plant homeodomain (PHD) motif, DNA-binding motifs, and SDI (Sdc1-Dpc-30 interaction) domains that provide docking sites for the final member of the WRAD complex, DPY-30. DPY-30 is a small nuclear and trans-golgi network protein whose worm homologue was first linked to X chromosome dosage compensation in *Caenorhabditis elegans* embryonic development [18, 19].

**Table 1:** KMT2C/D COMPASS complex components

A. Protein domains			
Domain	Interaction	Function	Protein Containing
**P**lant **h**omeo**d**omains (PHD)	Histones	Serve as epigenetic readers by identifying and binding to post-translationally modified histones.	KMT2C/D
**S**u(var)3-9, **E**nhancer-of-zeste, **T**rithorax (SET)	WARD Complex, Histone 3 tail	Catalyzes the transfers of a methyl group from S-adenosyl-L-methionine (SAM) donor factor to the ε amino group of recipient lysine residues.	KMT2C/D, EHMT1
**W**DR5 interaction **m**otif (WIM)	H3K4, WDR5	Arginine-containing motif which binds WDR5 (via Phe133 /Phe263 residues) and facilitates protein-protein interaction.	KMT2C/D
**K**abuki **i**nteraction surface (KIS)	H3K4, WRAD complex	KMT2 protein surface near the SET domain which combines with WRAD proteins to forms a secondary active site.	KMT2C/D
**W**DR5-**i**nteracting site (Win)	KMT2 proteins	Phenylalanine-containing site which binds KMT2 proteins via WIM. Also acts as H3R2me2 "reader" of poised genes.	WDR5
**W**DR5-**b**inding **m**otif (WBM)	RBBP5, NSL, NuRD, others	Facilitates binding of WDR5 to other proteins, including RBBP5 of the KMT2C/D COMPASS complex.	WDR5
**WD**40 motif (tryptophan-aspartic acid [W-D] dipeptide)	NA	Motif which forms a rigid circularized structure, facilitating protein-protein interactions in large protein complexes.	WDR5, RBBP5
**H**istone **a**cetyl**t**ransferase (HAT)	H3K27, others	Catalyzes acetylation of lysine residues. Histone tail lysine acetylation is generally linked with transcriptional activation.	CBP, P300
B. COMPASS associated proteins			
Protein	Interaction	Function	
**W**D repeat domain 5 (WDR5)	KMT2D, RBBP5	Part of WARD complex; acts as a histone specific reader associated with chromatin “poised” for transcription.	
**A**bsent, small or homeotic 2-like (ASH2L)	KMT2D, RBBP5, DPY30	Part of WARD complex; contains a histone-binding PHD and DNA-binding that provide docking sites for DPY-30.	
**R**etinoblastoma binding protein 5 (RBBP5)	WDR5, ASH2L	Part of WARD complex; acts as a scaffold protein between ASH2L and WDR5.	
**D**umpy 30 (Methyltransferase complex regulatory subunit)(DPY30)	ASH2L, DPY30	Part of WARD complex; small nuclear trans-golgi network protein involved in transcription, cell migration and endosomal trafficking.	
Activating signal cointegrator-2 (ASC-2)	KMT2C/D, RAR	Co-activates and helps recruit KMT2C/D to nuclear receptors.	
Creb binding protein (CBP)	UTX, RAR	Co-activates transcription factors and has intrinsic histone acetyltransferase activity.	
Enzyme p300 (EP300 or P300)	UTX, RAR	Co-activates transcription factors and has intrinsic histone acetyltransferase activity.	
PAX-interacting protein1-associated glutamate rich protein 1a (PA1)	PTIP	Stabilizes PTIP.	
PAX-interacting protein 1 (PTIP)	KMT2C/D, PA1	Involved in transcriptional regulation by binding various transcription factors, also has roles in DNA repair / progression through mitosis.	
Ubiquitously transcribed tetratricopeptide repeat, X chromosome (UTX/KDM6A)	KMT2C/D, RAR	Demethylates lysine 27 on histone 3, generally resulting activation of gene expression.	

Bolded letters indicate source of abbreviation. Shaded boxes in Table 1(B) denote WARD complex

*NA* not applicable, *Phe* phenylalanine, *NuRD* nucleosome remodeling deacetylase, *NSL* non-specific lethal, complex (also see text and List of Abbreviations)

#### WRAD-KMT interactions and activities

The WRAD complex couples with a region adjacent to the KMT2 SET domain (called the Kabuki interaction surface, KIS) to create a second active site that enhances di- and trimethylation of H3K4 residues [20]. The closely aligned surfaces of the ASH2L-RBBP5 dimer and KMT2 SET regions also each independently bind the required chemical co-factor s-adenosylmethionine (SAM). Together, WRAD and KMT2 proteins form a tight set of coordinated “pockets” that colocalize—(1) the SET catalytic domain, (2) the histone tail target, and (3) the methyl group-donating cofactor (SAM)—to facilitate histone tail methylation [21].

Comparative in vitro biochemical analysis of the six KMT2 proteins’ SET domain regions, however, shows some divergence in their methyltransferase catalytic activity and substrate specificity, both in isolation and when complexed with a recombinant WRAD complexes [22]. All WRAD/KMT2 SET complexes showed some degree of H3K4 monomethylation (H3K4me1) activity, with KMT2C-containing complexes being the least active. Methyltransferase activity against H3K4me1 substrate (leading to dimethylation, H3K4me2), however, was essentially absent for KMT2C and substantially weaker for KMT2D than other family members (KMT2A/B/F/G). Only KMTF/G (SET1A/B) demonstrated robust methylation of H3K4me2 residue (leading to trimethylation) [22]. Such intrinsic biochemical differences of the SET domain regions in KMT2 proteins support a divergent role for these genes in vivo (see “COMPASS complex biological function” section). Though SET domain region biochemical differences are one source of divergent function across the family, the KMT2 COMPASS complexes also diverge in activity and biological function as a result of their non-WRAD KMT2 complex binding partners.

#### KMT2 COMPASS complex subtypes

Mammalian KMT2 COMPASS complexes can be divided into three subtypes on the basis of their sequence and domain homology, as well as their non-WRAD complex binding partners. KMT2A and KMT2B COMPASS-like complexes are differentiated by their unique binding of the tumor suppressor protein Menin (encoded by *MEN1* gene) [23]. In contrast, KMT2F (SET1A) and KMT2G (SET1B) COMPASS complexes exclusively contain the cofactors WDR82 and the ASH2-binding protein CXXC1 and compose the second group [6]. In the third group, the KMT2C (MLL3/HALR) and KMT2D (MLL4/ALR) COMPASS-like complexes (K_CD_COMs, also historically known as ASC2-binding complexes, ASCOMs) bind the largest number of unique subunits (NCOA6/ASC2, KDM6A/UTX, PTIP, and PAGR1/PA1) (see below for full details) and, given their relationship to Kabuki syndrome (type 1 and 2) and type 2 Kleefstra syndrome, are discussed in greater detail below [24–26].

### K_CD_COM composition

Human K_CD_COMs were first isolated from HeLa cell nuclear lysates via immunoaffinity purification using an antibody against ASC2 (activating signal cointegrator-2/now known as NCOA6) [24]. Highly similar, large nuclear complexes (~ 2 megadaltons) were also later purified and characterized via alternative strategies (anti-KMT2D/ALR affinity purification [25] and anti-PTIP affinity purification [26]). In addition to KMT2C/D core proteins and the WRAD subunits, at least four additional complex members were reproducibly identified (Fig. 1b, Table 1(B)). These additional complex members have been extensively studied and are further detailed below.

#### Non-WRAD COMPASS complex members

Human K_CD_COMs contain at least four additional non-WRAD complex proteins, which add a diverse set of additional protein and DNA binding activities as well as histone modifying enzymatic activities (see Table 1(B)).

1. Nuclear receptor coactivator 6 (NCOA6) is a well-studied, essential nuclear receptor (NR)-interacting protein that both binds ligand-bound NRs (such as RAR, PPARγ, ERα, and others) and interacts with a broad array of coactivators including CBP/p300, NIF1, and multiple RNA-binding proteins (well-reviewed in [27, 28]).
2. PAX interacting factor 1 (PTIP) is a multifunctional nuclear protein with independent roles in double-stranded DNA repair (via binding of 53BP1) and transcriptional regulation via binding various transcription factors and K_CD_COMs [26, 29–31].
3. PAX-interacting protein1-associated glutamate rich protein 1a (PAGR1/PA1) is a stabilizer protein which tightly binds PTIP. PA1 is essential for early embryonic development and forms KMT2 COMPASS-independent PTIP-PA1 complexes with a special role in B cell class switch recombination [32, 33].
4. KDM6A (lysine demethylase 6A, also known as UTX, ubiquitously transcribed tetratricopeptide repeat, X chromosome) is a type 2 demethylase protein defined by its catalytic Jumonji C (JmjC) domain. The JmjC domain has an iron-containing [Fe(II)] active site and allows KDM6A to act as an α-ketogluterate-dependent oxygenase that removes di- and trimethyl groups from lysine 27 of histone 3 (H3K27me2/3) residues [34, 35]. A unique feature of K_CD_COMs is the inclusion of this second histone-modifying subunit (in addition to the lysine methylases KMT2C/D).
  Given KDM6A’s role as the second gene linked to Kabuki syndrome, this member of K_CD_COM has now been extensively studied. Encoded on the mammalian X chromosome, *Kdm6a* homozygous loss is embryonic lethal in females, whereas ~ 25% of *Kdm6a*-deficient male pups survive to adulthood, likely because of partial compensation by the Y chromosome paralog, *Kdm6c* (UTY) [36]. KDM6A has roles in multiple developmental processes, including posterior embryonic patterning and myogenesis [37, 38]. The KDM6A/UTX protein occupies specific homeobox (HOX) cluster gene promoters and increases their transcription while removing inhibitory H3K27me2/3 methylation marks [39]. Somatic mutations in *KDM6A* are linked to various cancers, and KDM6A/UTX has a role in cell cycle regulation by mediating demethylation of retinoblastoma-binding proteins [40, 41]. The extent to which these ascribed cellular functions are dependent or independent of KDM6A’s direct interaction with the of K_CD_COM remains unclear; however, more extensive biochemical characterization of the COMPASS complex suggests that some binding partners may be more dynamic than previously recognized.

#### KMT2 COMPASS complex heterogeneity

The six known mammalian KMT2 COMPASS complexes are uniquely defined by their core proteins, and their various subunits do not exclusively bind to KMT2 proteins. Multiple mass spectrometry-based proteomics studies have shown that the WRAD proteins are at least four times more abundant than all known KMT2 core proteins combined [42]. WDR5 binds ~ 200 different proteins, and DPY-30 is a member of the NURF chromatin remodeling complex, as well as the COMPASS complex. The K_CD_COMs’ non-WRAD subunits are also more abundant than the KMT2 proteins and likewise are promiscuous in their protein interactions. Of note, the PTIP subunit is more abundantly found as a 53BP1-complex member or as an independent heterodimer complexed with PA1 than as a member of K_CD_COM [32, 42]. In addition, substoichiometric levels of NCOA6 and KDM6A protein were detected in HeLa cell K_CD_COM, suggesting that fewer than half of these complexes contained these specific subunits at steady-state. Experimental evidence supports dynamic, context-dependent binding of certain COMPASS subunits to the complex, such as the NCOA6/ASC2 subunit that acts as an adapter for nuclear receptors (with or without ligands) and/or transcription factors [28]. The heterogeneity of cellular K_CD_COMs (i.e., with or without NCOA6 or KDM6A) and the extent to which this diversity is specific to cell lineage, differentiation state and activation state are currently undefined.

### COMPASS complex biological function

KMT2 proteins function as (1) histone methyltransferases and (2) chromatin-bound protein scaffolds. H3K4 methylation is heterogeneous and dynamic, with H3K4me1 being most closely associated with enhancer elements and active gene bodies, whereas H3K4me3 marks decorate active promoters. The relative contributions of the three different KMT2 COMPASS complexes to maintaining genome-wide and gene-specific H3K4me1, H3K4me2, and H3K4me3 appear to vary. KMT2F/G COMPASS complexes preferentially deposit “bulk” H3K4me2 and H3K4me3 at promoters and through gene bodies of actively transcribed genes [43]. KMT2A/B COMPASS complexes also deposit H3K4me3 marks, but these similar genes nevertheless have non-redundant roles with more tightly defined regulatory functions. Specifically, KMT2A COMPASS has a key role in regulating the HOX gene clusters [44], whereas KMT2B COMPASS has a special role in maintaining specific promoter types (bivalent H3K27me3^+^/H3K4m3^+^) [45]. In contrast, the K_CD_COMs have emerged as the major H3K4 monomethylases of active enhancers in the mammalian genome [46–48](see “KCDCOMs, p300/CBP, and enhancer regulation” section); however, earlier work also supports a role for the ASCOMs in placing H3K3me3 marks on the promoters of nuclear receptor-regulated genes (via NCOA6/ASC-2 interactions) [28, 49, 50]. From this point onward, we will focus on the emerging role of KMT2C/D complexes in the priming of *differentiation*-*inducing* enhancers during cell fate transitions and their putative relationship with human disease [48, 51, 52].

### K_CD_COMs, p300/CBP, and enhancer regulation

The identification of the major Drosophila histone monomethylase gene, trithorax-related (*trr*), initiated foundational biochemical and functions studies of *trr*’s mammalian homologues, *KMT2C* and *KMT2D* [53]. The partially redundant roles of KMT2C and KMT2D as H3K4 monomethylases emerged via both in vitro cellular studies and in vivo mouse models. Study of the human colorectal carcinoma cell line HCT116, which incidentally harbors homozygous *KMT2C* (*MLL3*)-inactivating mutations, facilitated the first genome-wide characterization of KMT2D chromatin binding sites [54]. By additional genetic disruption of *KMT2D* (*MLL4*), double-knockout cells (HCT116-MLL3^ΔSET^/4^ΔSET^) were generated and shown to have bulk reduction in total H3K4me1 [55]. Chromatin immunoprecipitation sequencing (ChIP-Seq) studies using both HCT116-MLL3^ΔSET^/4^ΔSET^ cells and mouse embryonic stem cells (mESCs) showed colocalization of KMT2D and the E1A-binding protein p300 (EP300 or p300), a well-established marker of active enhancers and a potent histone acetylase [47]. As expected, overlapping KMT2D/p300 peaks colocalized with H3K4me1and histone 3 lysine 27 acetylation (H3K27ac) marks [47]. These studies established K_CD_COMs as major enhancer coactivators during mammalian development.

Genetically engineered mouse studies have also supported the role of K_CD_COMs as critical enhancer primers during cellular transitions. Utilizing gene trap approaches (global nulls) [46], conditional knockouts (Cre-targeted deletion) [46], and a SET domain targeting system [49, 56, 57], the functional redundancy of KMT2C and KMT2D have been extensively explored in a variety of developmental contexts. Early interest in the mechanisms linking the of K_CD_COMs to PPARγ-driven adipocyte differentiation led to the generation of Mll3^Δ/Δ^ (*Kmt2c*) mice (bearing homozygously disrupted SET domains), which were found to have reduced white fat production and poor survival unless maintained on a mixed genetic background [49, 50]. Global *Kmt2c* and *Kmt2d* knockout mice were later found to suffer from embryonic or perinatal lethality, and thus a *Kmt2d* conditional knockout system was subsequently developed. Conditional deletion of *Kmt2d* in brown adipose tissue and skeletal muscle (via *Myf5*-*Cre*) resulted in impaired adipogenesis and myogenesis, but this effect was not seen in *Kmt2c* knockout animals, highlighting the critical non-redundant role of KMT2D in these specific differentiation programs. Utilizing in vitro differentiation protocols and ChIP-Seq, KMT2D was shown to preferentially bind active promoters in a cell lineage- and differentiation stage-specific manner, as well as colocalize with lineage-determining transcription factors (e.g., C/EBPβ and MyoD) and epigenetic markers of active enhancers (H3K3me1/2 and H3K27ac) [46]. Similarly, conditional deletion of *Kmt2d* from embryonic heart tissue dysregulated a broader array of genes earlier in development, but the absolute number of dysregulated genes declined over developmental time [58]. Furthermore, KMT2D ChIP-Seq of mESC-derived cardiomyocytes revealed preferential binding to distal regulatory regions enriched for specific transcription factor binding motifs (e.g., HIF1A TEAD1, SRY). Approximately 16.5% of these KMT2D sites showed reduced H3K4me1/2 marks with KMT2D deletion, but surprisingly few KMT2D-bound genes showed reduced levels of gene expression (~ 1%), suggesting a more restricted role for KMT2D in commissioning rather than maintaining enhancer activity following terminal differentiation [58].

Further evidence of KMT2D’s specific role in *de novo* “commissioning” of enhancers emerged with in-depth investigations of functional interactions between K_CD_COMs and p300 (and/or its highly similar family member CREB binding protein [CREBBP or CBP]). In *Kmt2c*^−/−^ (*Mll3*^−/−^) knockout mESCs, KMT2D was shown to bind active enhancers (AE, marked with H3K4me1/H3K27ac) and to colocalize with the enhancer-activating protein p300. Surprisingly, in mESCs, only a modest number of KMT2D-bound AE-associated genes had reduced baseline expression with the additional depletion of KMT2D (i.e., KMT2C/D double knockout). In contrast, when KMT2C/D double-knockout mESCs were differentiated into adipocytes, they demonstrated profoundly impaired recruitment of p300 to de novo enhancers and reduced target gene expression [46]. These results demonstrate a specific role of K_CD_COMs in the initial commissioning of select enhancer elements but not an ongoing requirement for K_CD_COMs to maintain enhancers once activated. The generation of methylase activity-deficient double-mutant KMT2C^Y4792A^/KMT2D^Y5477A^ mESCs further revealed that KMT2C/D proteins possess a critical non-enzymatic role in enhancer regulation, acting as a coactivator to recruit and regulate RNA polymerase II binding at enhancer elements but not promoters [59]. Furthermore, during retinoic acid signaling, this coactivation occurs via KDM6A-mediated physical coupling of p300/CBP to the rest of the KMT2D complex [52]. In summary, a molecular model is emerging of closely coordinated interaction between the K_CD_COMs and p300/CBP to commission select enhancers during specific cell fate transitions. These new insights provide a framework for understanding the molecular mechanisms leading to the diverse and overlapping phenotypic manifestations of Kabuki, Rubinstein-Taybi, and Klefstra type 2 syndromes.

## K_CD_COM-associated diseases (K_CD_COM-ADs)

### Kabuki syndrome

Kabuki syndrome (KS) is a multi-systemic, congenital disorder characterized by distinct facial features, infantile hypotonia with persistent developmental delay, intellectual impairment and a diverse array of additional associated features with variable degrees of expressivity [60–64] (see Table 2). The frequency of clinical KS is estimated to be at least one in 32,000 live births [62], but recent clinical experience in the post-exomic sequence era supports a rate as frequent as one in 10,000 live birth (personal communication, S. Banka MBBS, PhD).

**Table 2:** Clinical features of K_CD_COM-associated diseases

	KABUKI syndrome [60, 61, 65]	RUBENSTEIN-TAYBI syndrome [66–69]	KLEEFSTRA syndrome [70–72]
Gene(s)	*KMT2D* (Type 1)	*CBP* (Type 1)	*EHMT1* (Type 1*)
	*KDM6A* (Type 2)	*EP300* (Type 2)	*KMT2C* (Type 2)
Growth	Post-natal growth restriction	Post-natal growth delay with subsequent excessive weight gain during childhood	Markedly reduced linear growth and head circumference. Normal, reduced or increased weight.
Craniofacial features			
Head size	Variable microcephaly	Microcephaly	Brachycephaly
Eyes	Long palpebral fissures with eversion of the lateral one-third of the lower eyelid	Down-slanting palpebral fissures, long eye lashes	Hypertelorism
Eyebrows	Arch eyebrows with notching/sparseness of the lateral portion	Highly arched eyebrows	Synophrys (long, single eye brow)
Nose	Short columella with depressed nasal tip	Broad nasal bridge, beaked nose and prominent nasal septum extending below nares	Anteverted nares, macroglossia
Mouth/Lips	Tented upper lip	Small opening of mouth	Tented cupid’s bow portion of upper lip
Palate/Jaw	High arched palate with variable cleft, tooth abnormalities	High-arched palate with micrognathia	Prognathism
Ears	Large prominent cupped ears	Low set auricles	Thick ear helices
Cognitive and behavioral features	Mild to moderate intellectual disability with relative strength in verbal comprehension and more pronounced deficiencies in perceptual and nonverbal skills. Language and speech defects with expressive (dysarthria and dyspraxia) and expressive deficits.	Typically, moderate intellectual disability, ranging from mild to profound with relative weakness in working memory. Behavior characterized by high sociability but with challenges in communication, impulsivity, distraction, anxiety, and obsessive-compulsive attributes. Patients may also have mood disorders, tics and autistic behavior (more frequently in patients with CBP mutation vs EP300 mutation).	Mild, moderate or severe intellectual disability, language and delays, autistic behaviors, disordered sleeping, aggressive/hyperactive behaviors and auto-mutilatory behaviors
Other features	Skeletal anomalies (brachymesophalangy, brachydactyly V, vertebral defects, 5^th^ digit clinodactyly) and dermatoglyphic abnormalities (persistent fingertip pads), seizure disorders. Hypogammaglobulinemia, autoimmunity	Small opening of mouth	Skeletal features: kyphosis and scoliosis. Seizure disorders

#### Clinical phenotype

First described in 1981 by two independent Japanese clinical geneticists [60, 61], KS (or Niikawa-Kuroki syndrome) is classically defined by five cardinal features: (1) postnatal growth restriction, (2) distinct facial dysmorphic features (long palpebral fissures with eversion of the lateral one-third of the lower eyelid; arched eyebrows with notching/sparseness of the lateral portion; large, prominent, cupped ears; and short columella with depressed nasal tip, craniosynostosis), (3) skeletal anomalies (brachymesophalangy, brachydactyly V, vertebral defects, fifth digit clinodactyly), (4) dermatoglyphic abnormalities (persistent fingertip pads), and (5) intellectual disability (see below for further discussion) [62]. Efforts to refine the diagnostic criteria for KS have emerged over the last 5 years following the identification of the genetic etiology for most cases (see below) [65]. Stricter application of existing KS criteria or the use of phenotypic scoring systems (e.g., MLL2-Kabuki score) have been proposed to improve the sensitivity and specificity of clinical KS diagnosis, given the phenotypic heterogeneity and overlap seen between KS and other defined genetic conditions [73, 74]. To date, there is no consensus pathognomonic physical feature(s) for KS and instead, most experts agree that the diagnosis should be made on the basis of a combination of phenotypic and genetic findings [75].

#### Genetic etiology

Autosomal dominant mutations in KMT2D (OMIM 147920) were first linked to KS in 2010 [76], and this was later designated as type 1 KS (KS1). Two years later, an X-linked dominant form of the diseases was established with discovery of pathogenic mutations in KDM6A (OMIM 300867) (type 2 KS [KS2]) [77]. The genetic analysis of larger KS cohorts has now established that approximately 43–76% of clinical cases are linked to KMT2D mutations (KS1), ~ 1–6% of cases are associated with KDM6A mutations (KS2) and ~ 24–57% of cases remain genetically undetermined [64, 73]. Genetic mosaicism has also been described for KS1 [78], as well as vertical transmission (dominant inheritance) of pathologic KMT2D/KDM6A mutations from mother to patients [64, 79, 80]. Nevertheless, the vast majority of genetically defined KS cases appear to be patient-specific, de novo mutations [64, 81].

The majority of KS1- and KS2-linked mutations are categorized as “truncating” (nonsense, splice site, or insertion/deletion events) mutations that are predicted to disrupt gene expression and likely to induce pathology via haploinsufficiency. Locus genomic aberrations (deletion/duplication/rearrangements) and splice site mutations occur more often in the *KDM6A* than *KMT2D* locus, with splice site mutations being three times more frequent [64]. In both KS1 and KS2, in-frame missense mutations are less common than the truncating subtypes listed above, with recent analyses revealing a rate of 16–33% in *KMT2D* (KS1) and 7.5% in *KDM6A* (KS2) [64] [82]. In both genes, missense mutations occurred most frequently in undefined regions lacking currently defined functional domains. A recent study, however, has shown that the domain-rich KMT2D C-terminal region, where the FYR protein-protein interaction domain and catalytic SET domains are located, harbored 36% of the disease-related missense mutations, and subsequent functional analysis showed that nine out of 14 investigated mutations had altered H3K4 methylation activity [82].

#### Neurologic/behavioral phenotype

Mild-to-moderate intellectual disability is a cardinal feature of KS, with an early, well-powered study reporting an average full scale intelligence quotient (FSIQ) of 62 (range 30–83, *n* = 62) in clinically diagnosed subjects [62]. This result was recently confirmed in two independent cohorts of patients with genetically confirmed KS (average FSIQ 57, range 40–103 [83], average FSIQ 67, range 25–109 [84]). Of note, more in-depth neuropsychiatric testing in subjects with KS has shown discordance within cognitive domains, with relative strength in verbal comprehension and more pronounced deficiencies in perceptual/nonverbal reasoning and processing speed [83, 85]. A similar recent study utilizing IQ-matched non-KS control subjects confirmed visual motor, visual perception, and visual motor memory defects in KS patients while language function was less affected [86]. Language and speech defects are also common in subjects with KS, with both expressive (dysarthria and dyspraxia) and receptive deficits described [87, 88]. A recent study of patients with genetically confirmed KS showed a heterogeneous pattern of oromotor, speech, and language deficits, consistent with the variable degree of orofacial, hearing, and cognitive defects seen in patients with KS [89].

In addition to mild-moderate intellectual disability, infantile hypotonia is highly prevalent in subjects with KS, affecting between 51 and 98% of patients [63]. Despite general resolution of this phenotype with age, gross motor skills are delayed in most patients with KS, which may have an impact on adaptive functioning during activities of daily living [84]. In contrast to the more commonly observed intellectual disability and hypotonia, a smaller subset of patients with KS also develop seizure disorders, with most studies reporting a prevalence ranging from 5 to 16% [90]. Although postnatal microcephaly occurs in 29–56% of subjects with KS, most reports state that specific central nervous system (CNS) anatomic lesions are not observed via imaging, even in patients with KS with seizure disorders [90–92]. KMT2D’s high expression in the dentate gyrus and cerebellum and the gene’s proposed role in neuron differentiation support a direct mechanistic connection between at least some aspects of the KS neuropsychiatric phenotype and loss of gene expression in CNS tissues [85, 93].

### Rubinstein-Taybi syndrome

#### Clinical phenotype

Rubinstein-Taybi syndrome (RTS) (or broad thumb-hallux syndrome) is a rare congenital syndrome first reported by Jack Rubinstein and Hooshang Taybi in 1963 [94]. The RTS phenotype is characterized by a distinct amalgamation of several major features including (1) postnatal growth restriction, (2) intellectual disability with stereotyped behaviors, (3) characteristic physical features (especially facial and limb defects), and (4) increased risk for childhood tumors (see Table 2) [66]. Core dysmorphic features include prenatal and postnatal growth restriction, microcephaly, and several distinct facial and limb anomalies. Facial dysmorphisms (especially of the nose/eye) and abnormal facial expressions are commonly reported. Ocular features include long eyelashes, highly arched eyebrows, and down-slanting palpebral fissures. Nasal features manifest as broad nasal bridge and prominent nasal septum extending below nares. Patients with RTS often have a high-arched palate with micrognathia [94, 95]. Distinct facial expressions are also classically associated with RTS, including grimace or prominent smile with closing eyes. Limb abnormalities include broad thumbs with radial deviation and broad first toes with medial deviation. Partial duplication of digits and terminal broadening of phalanges has also been observed. Patients commonly display hirsutism [66, 94, 96, 97]. Patients typically have delayed fetal and infantile growth with subsequent excessive weight gain during childhood. RTS is uncommon, with an estimated prevalence of approximately one in 100,000 to 125,000 newborn infants [66, 98].

The RTS phenotype is heterogeneous and affects multiple organ systems. In the perinatal/neonatal period, a third of patients with RTS are born from pregnancies complicated by polyhydramnios, four-fifths have feeding difficulties, half experience respiratory problems, and over a third have tear duct obstruction [66]. Growth retardation (< 3^rd^ percentile) occurs in 75% of patients [99]. Increased susceptibility to upper airway infections occurs in 60% of patients. Other common medical complications include ocular problems (strabismus in 58% and/or refractive error in 41% of patients) and congenital heart defects (mostly patent ductus arteriosus, ventricular septal defect, or atrial septal defect). Less common medical problems include hearing loss (24%), keloid formation (25%), seizures (23%), and benign or malignant tumors (29%) [67, 68]. Due to the wide array of potential medical problems, some providers recommend one or more baseline evaluations in patients with RTS, including electrocardiogram, echocardiogram, renal ultrasound with consideration for voiding cystourethrogram, and hearing evaluation [69, 100]. Evaluation by a pediatric ophthalmologist, pediatric cardiologist, and/or pediatric nephrologist may be necessary, depending on a particular patient’s presentation [66].

A recent population-based study of a Dutch RTS cohort evaluated characteristics of benign and malignant tumors in patients with RTS with mutations in CREBBP (70%) and EP300 (5–10%). Tumors reported include pilomatricoma, hemangioma, breast carcinoma, nevi, dermatofibroma, breast fibroadenoma, colon carcinoma, high-grade squamous intraepithelial lesion of cervix, non-small cell lung carcinoma, and diffuse large-cell B cell lymphoma [100]. Thirty-five benign and malignant tumors were reported in 26 of 87 individuals in the study, with meningiomas and pilomatricomas being the most frequent benign tumors. Five malignant tumors were observed in four individuals with RTS, including medulloblastoma, diffuse large-cell B cell lymphoma, breast cancer, non-small cell lung carcinoma, and colon carcinoma. However, no clear genotype-phenotype correlation was evident from the study, and the small number of patients in the cohort was a limitation to fully understanding the risk of tumors in patients with RTS [100].

Because recurrent respiratory infections are a common problem in RTS, characterization of the humoral immune system was performed in a Brazilian cohort. A study by Torres et al. indicated that patients with RTS had normal or elevated serum immunoglobulin levels, normal salivary IgA levels, and robust antibody response to both protein and polysaccharide antigens. However, most patients in the cohort had high serum IgM levels and high number of total B cells. Patients were noted to have B cell subsets with a high percentage of apoptosis (measured by flow cytometry of peripheral blood samples stained with annexin V FITC (fluorescein isothiocyanate) and propidium iodide-PE using an apoptosis detection kit), suggesting dysregulation in the B cell compartment [101]. Studies in mice indicate that individual loss of CBP or p300 is surprisingly well tolerated and not essential for B cell development and function past the pro-B cell stage. However, B cells lacking both CBP and p300 were rare to nonexistent, which indicates that, together, these coactivators play a crucial role in B lymphocyte development and function that is not redundant in other transcriptional cofactors such as GCN5 or P/CAF that share similar function (e.g., protein acetyltransferase activity) [102]. These observations may provide insight into why patients with RTS also have increased susceptibility to infections, particularly respiratory infections, during infancy and childhood [103].

#### Genetic etiology

RTS is often noted as one of the first described “disorder of multiple congenital anomalies.” Nevertheless, identification of the first RTS-linked gene occurred 32 years after the syndrome’s initial description. Cytogenetic analyses of patients with RTS in the early 1990s suggested alterations involving chromosome 16p13.3. Subsequent gene mapping and cohort sequencing efforts linked over 50% of RTS cases to deletions/mutations in the cAMP response element-binding protein (CREB)-binding protein (CREBBP or CBP) gene (OMIM 180849) (termed RTS type 1, *RSTS1*) [68, 104, 105]. The genetic etiology of RTS, however, has proven heterogeneous with the later identification of RTS-linked mutations in the CBP paralog E1A binding protein p300 (EP300 or p300) located on chromosome 22q13.2 (OMIM 613684, termed RTS type 2, *RSTS2*) [106]. Patients with RTS2 only represent ~ 3% of patients with RTS [106, 107], and thus, the genetic etiology of the remaining clinically diagnosed cases remains unclear. RTS typically occurs as a sporadic de novo mutation or chromosomal deletion, although vertical transmission via an autosomal dominant inheritance pattern has also been observed. To date, there are currently no standardized diagnostic criteria for RTS, but the clinical diagnosis is made via a combination of supportive phenotypic findings, chromosome analysis using fluorescence in situ hybridization (FISH) to probe for deletions of chromosome 16p13.3, and sequencing analysis for mutations in CBP and p300 [66, 104, 108].

Genotype-phenotype correlations have been investigated in RTS [68]. In a cohort of 93 patients diagnosed with RTS, mutation analysis was performed on all 31 coding exon and exon-intron junctions of CBP. FISH analysis was performed on a subset of patients for large deletions. There were 64 unique genetic variants observed, with definitive pathogenic mutations in 52 patients (56%). Four broad categories of CBP mutations types were observed—truncating mutations, large deletion mutations, missense mutations, or no CBP mutation. Mutations detected included 36 truncating or splice-site variant mutations (56% of mutations), six large deletions detectable by FISH (9% of mutations), ten missense mutations (16% of mutations), and 14 variants of unknown significance (22% of mutations). The majority of mutations affected the histone acetyltransferase (HAT) domain of CBP or predicted protein termination prior to the HAT region. Extensive phenotypic data were collected. All four groups displayed distinctive facial and thumb dysmorphisms. There was a trend toward lower intelligence quotient (IQ) and increased autistic features in patients with large deletions. Phenotypes associated with the four different mutation types otherwise had subtle differences.

In a cohort of 52 patients with *EP300* mutations [67] and interstitial deletions (51 different alterations), there were three interstitial deletions (6%), 15 nonsense mutations (29%), 20 frameshift mutations (43%), four splice-site mutations (8%), three in-frame deletions (6%), three missense mutations (6%), and one variant of unknown significance (2%). Of these mutations, 11 (22%) were associated with maternal pre-eclampsia, including two interstitial deletions, one nonsense mutation, four frameshift mutations, three splice site mutations, and one in-frame deletion. Although mutations were found throughout the entire distribution of *EP300*, there were two particularly large clusters of nonsense mutations at the HAT and kinase-inducible domain interacting (KIX) domains. There were no detectable, statistically significant correlations between the mutation location and patient phenotype. However, as the HAT domain is highly conserved between CBP and p300, it is possible that missense mutations involving the HAT domains of both genes (*CBP*, *EP300*) lead to typical RTS dysmorphisms and intellectual disability. However, to date, no data exist to support this conjecture.

In comparing patients with mutations in *CBP* (*n* = 308) and *EP300* (*n* = 52), there is no significant difference in observed rates of postnatal growth retardation, overall rate of intellectual disability, nor several of the anatomical abnormalities (e.g., cardiovascular anomalies, urinary tract anomalies, scoliosis, obesity and presence of keloids). Autistic behavior, hand anomalies, and some of the facial dysmorphisms, particularly the ocular, nasal, and oral anomalies, are more frequently found in patients with *CBP* mutations than *EP300* mutations. However, many facial abnormalities are not significantly different between patients with *CBP* and *EP300* mutations [67]. The similarities of RTS phenotypes between the various types of mutations suggest that the multiple genes affiliated with RTS etiology likely have effects through a common pathway (see “Overview” section).

The heterogeneous genetic mutations in *CBP* and *EP300* result in subsequent epigenetic consequences, which ultimately manifest as RTS disease phenotype. Multiple genetic models have been proposed to explain the ensuing disease phenotype. It has been observed that truncating mutations, in addition to null mutations, can result in RTS. Patients with microdeletions of one copy of the entire *CREBBP* gene support a haploinsufficiency model for CREBBP effects [104]. This finding implies that two functional copies are necessary to produce sufficient protein, thus supporting a haploinsufficiency model of disease [109]. Another interesting observation is that injection of a CBP-CREB binding domain (KIX domain) into fibroblasts inhibits transcriptional activation of the *Cre*-*LacZ* reporter gene. This indicates that an aberrant protein product derived from the mutant allele inhibits the wild-type protein, thus supporting a dominant-negative model of disease. Truncated protein products have been found by Western blot, with confirming sequence analysis in patients with RTS [104, 109, 110].

#### Molecular function

CBP and p300 are large, related multi-domain nuclear proteins that function as coactivators (via interactions the K_CD_COMs), as well as cofactors, for a large number of transcriptional regulators. CBP is generally highly conserved throughout phylogeny, with ~ 95% homology between human and mouse [104, 109]. CBP binds the well-studied transcription factor CREB and acts as a coactivator to promote gene expression. CBP’s protein-interacting domains facilitate the protein’s regulation of transcriptional machinery. As previously discussed, CBP and p300 also harbor the catalytic HAT domain, which facilitates histone acetylation (H3K27ac). Intrinsic CBP HAT activity is necessary for CREB-mediated gene expression. The HAT activity of CBP modulates gene expression by acetylation of histones H3 and H4. Acetylated H3 and H4 serve to decondense chromatin and allow for enhanced gene transcription. CREB-dependent transcription plays vital roles in growth, development, and long-term memory formation in mice [109, 111]. CBP also recruits transcriptional machinery, including the RNA polymerase II complex, coactivators, and repressors to bind enhancer elements. Another function of CBP is regulation of histone methyltransferase expression, which leads to gene silencing through hypertrimethylation of histone H3 (H3K9me3) and pericentromeric heterochromatin condensation [112].

In addition to acetylating histone tail substrates, CBP also has a major role in acetylating the tumor suppressor P53. Damaged DNA results in p53 activation via phosphorylation by a variety of kinases and acetylation at specific amino-acid residues by CBP and p300 [113]. P53 acetylation is thought to promote activation of target genes by increasing the stability of p53-p300-DNA complex. One hypothesis for why patients with RTS have increased incidence of childhood cancers is that mutated CBP likely has less acetylation activity against the tumor suppressor activity of p53, thus increasing the risk of tumorigenesis [114].

#### Neurologic/behavioral phenotype

Our understanding of developmental and behavioral outcomes in individuals with RTS is limited by a number of factors. Many older studies and studies with parent-report measures may be limited by a lack of genotyping of the participants. Older studies and even more recent larger studies often rely on a clinical diagnosis of RTS. The educational and therapeutic context across time and countries can also contribute to outcomes. Shifting cultural norms of community integration and a therapeutic focus on modifiable aspects of adaptive functioning and development over time may prompt variability in outcomes within populations of individuals with RTS.

In general, many studies have shown a range of cognitive abilities from profound intellectual disability to borderline cognitive functioning (< 25–79) [115]. More recent studies with larger cohorts of children note an average of cognitive or adaptive abilities that is in the moderate range of disability (mean IQ of 48.5 [116], mean adaptive composite score of 43.6 [117]).

Difficulties in working memory have been described by Waite et al. (2016) [118]. This study compared 32 children with RTS to “mentally matched,” typically developing children. They found that the group of children with RTS had more challenges in working memory span, suggesting a relative weakness in individuals with RTS. In contrast, visuo-spatial memory in children with RTS was similar to mentally matched, typically developing children.

An understanding of behavioral phenotype among individuals with RTS is limited by the available evidence and biases within existing papers. The behavioral phenotype of individuals with RTS has described high sociability but with difficulties in communication, impulsivity, distractibility, anxiety, and obsessive-compulsive features [115]. Among 13 individuals with RTS seen within a psychiatric care setting, diagnoses of mood disorders, tic disorders/obsessive-compulsive disorder spectrum, and autism spectrum disorders were noted [119].

In a more recent study of anxiety-related disorders in syndromes including RTS [120], 27 individuals with RTS noted a relatively high rate of obsessive-compulsive disorder (40%) and panic disorder (37%). In most categories of anxiety disorders, males were more commonly reported to have anxiety disorders than were females.

Moss et al. evaluated 27 individuals with RTS and comparison groups of other syndromes through caregiver report using the Social Sociability Questionnaire for People with Intellectual Disability [117]. As compared to other syndromes (Down syndrome, Angelman syndrome, and Fragile X syndrome), individuals with RTS had the highest prevalence of extreme sociability with unfamiliar people. Individuals with RTS had a high level of sociability in familiar and unfamiliar social situations.

### Kleefstra syndrome

#### Clinical phenotype

Kleefstra syndrome (KSS) is a heterogeneous, genetic, neurodevelopmental disability disorder characterized by intellectual disability and an array of distinct clinical manifestations. Intellectual disability ranges from mild to severe, with many patients having a moderate intellectual disability. Virtually all patients with KSS have language and motor delays. They often have behavioral problems manifested as autism or traits resembling autistic behavior. Some patients have disordered sleeping, aggressive/hyperactive behaviors, and automutilatory behaviors. Craniofacial dysmorphisms include brachycephaly, synophrys (long, single eye brow), anteverted nares, tented cupid’s bow portion of upper lip, everted lower lip, prognathism, and macroglossia. Patients typically have markedly reduced linear growth and head circumference with normal, increased, or decreased weight. The majority of patients have hypotonia, and many have seizure disorders. Patients often have skeletal abnormalities including kyphosis and scoliosis. Conotruncal heart defects and skin depigmentation have also been observed [70].

#### Genetic etiology

Characterization of KSS arose from recognition of a characteristic and clinically identifiable phenotype involving variable intellectual disability, hypotonia and craniofacial dysmorphisms in a cohort of 30 patients who shared submicroscopic subtelomeric deletions of chromosome 9q [121]. The minimal region responsible for the 9q subtelomeric deletion phenotype (9q^−^) was initially estimated to be around 1 Mb [122] and subsequently reduced to around 700 kb that contained at least five candidate genes (*ZMYND19*, *ARRDC1*, *C9ORF37*, *EHMT1*, and *CACNA1B*) and narrowed the scope of deletion to 9q34.3 [121]. Further work, particularly from characterization of breakpoints in a female with balanced translocation t(X;9)(p11.23;q34.3), indicated that the chromosome 9 breakpoint disrupted the *EHMT1* gene in intron 9. Mutation analysis in patients with a clinical phenotype reminiscent of 9q subtelomeric deletion syndrome lead to evidence for a causative role of *EHMT1* haploinsufficiency as a genetic etiology of KSS, and these patients are designated as having KSS type 1 (KLEFS1) [71]. *EHMT1* encodes a protein that regulates gene expression through histone 3 lysine 9 dimethylation (H3K9me2) [71]. This is generally considered to be a repressive histone mark in euchromatic regions of the genome.

In spite of identifying *EHMT1* as a causative gene of KSS, several individuals that are *EHMT1* mutation negative, but with phenotypes resembling the core features of KSS, have also been observed. Genetic analyses of such individuals have identified de novo mutations in four genes, including *MBD5*, *SMARCB1*, *NR1I3*, and *KMT2C*. All four of these genes encode proteins associated with regulation of gene expression and/or chromatin structure [72]. By application of large-scale, whole-exome sequencing in a series of patients with unexplained neurodevelopmental disorders, *KMT2C* was identified as the genetic etiology of several individuals with the KSS phenotype. These individuals are designated as having KSS type 2 (KLEFS2). A cohort of six individuals with the KSS phenotype and *KMT2C* mutations gave insight into the nature of the mutations. The mutations of all of these individuals are predicted to result in loss of function of KMT2C, although no functional studies of patient cells have been performed. The mutations involve five unique heterozygous frameshift or truncating mutations in *KMT2C* and one patient with a de novo heterozygous 203-kb intragenic deletion in *KMT2C* [70].

#### Molecular function

In humans, *KMT2C* is on chromosome 7q36 and encodes histone-lysine *N*-methyltransferase 2C, which monomethylates lysine 4 of histone H3 (H3K4me1), thus leading to transcriptional activation. The molecular function of *KMT2C* has been explored by studying its Drosophila ortholog, *trr*, as well as *G9a*, the Drosophila ortholog of *EHMT1*. *trr* encodes a histone methyltransferase that facilitates transcriptional activation through monomethylation and trimethylation of histone H3 at lysine 4 (H3K4me1 and H3K4me3). trr is orthologous to human KMT2C and KMT2D and acts in a conserved COMPASS-like protein complex to deposit H3K4me1 at enhancers. trr also interacts with ecdysone (an insect steroidal prohormone of the major molting hormone) receptor and is a coactivator of ecdysone-mediated transcription by performing H3K4me3 at promoters. *G9a* encodes a histone methyltransferase that facilitates transcriptional repression through monomethylation and dimethylation of histone H3 at lysine 9 (H3K9me2), analogous to EHMT1 in humans [70].

In order to study the functional role of KMT2C in neurons and thus infer how *KMT2C* mutations may be implicated in the intellectual disability syndrome phenotype, studies were conducted in Drosophila *trr*, which shares a one-to-two evolutionary relationship with human orthologs *KMT2C* and *KMT2D*. Because homozygous mutations in *trr* are lethal, a Gal4/UAS system and inducible RNA interference (RNAi) system were employed to ascertain the role of trr in the adult fly nervous system. Studies indicated that trr is localized in the nuclei of the mushroom body calyx (structures in fly brain required for olfactory learning and memory). Though flies with *trr* knockdown had no gross morphologic defects in the mushroom body, they did demonstrate abnormal courtship conditioning memory (a classic and well-studied behavior paradigm), which indicates a defect in learning and memory. Chromatin immunoprecipitation combined with next-generation sequencing (ChIP-seq) studies of wild-type Drosophila heads with antibody to trr revealed genomic regions that were highly enriched for trr binding. Analysis of these regions indicated that approximately 75% of trr binding sites were located within 1 kb upstream or downstream of transcription start sites (tss) of 2362 unique genes and that approximately 25% of trr binding sites were associated with other genomic features, such as low-complexity regions or transcription termination sites. These findings are consistent with the roles of KMT2C and KMT2D in transcriptional activation [70, 123].

KSS types 1 and 2 (KLEFS1 and KLEFS2) share a common link of being resultant syndromes from genetic defects involving histone methyltransferases. However, KLEFS1 is linked to mutations in H3K9 methyltransferase EHMT1 (i.e., KMT1D), which is in a different group of methyltransferases and does not share cofactors with KMT2C or KMT2D. We refer the reader to other review articles for more information on the epigenetic and cellular mechanisms associated with EHMT1 (see [123]an d[124]).

## Phenotypic overlap IN K_CD_COM-ADs

### Overview

As outlined above, at least five recognized human developmental syndromes currently are linked to germline mutations in K_CD_COM proteins (KMT2C/D, KDM6A) or associated chromatin-activating molecules (p300/CBP). The genetic and molecular links between these diseases are further strengthened by the substantial phenotypic overlap noted in subjects diagnosed with these syndromes (see Table 2) [125]. In addition to prominent intellectual impairment and behavioral abnormalities, key structural defects also occur with high frequency across all three conditions, including short stature, skeletal abnormalities, cardiovascular defects, and craniofacial defects, which are further detailed below.

### Craniofacial development

**Fig. 2:**
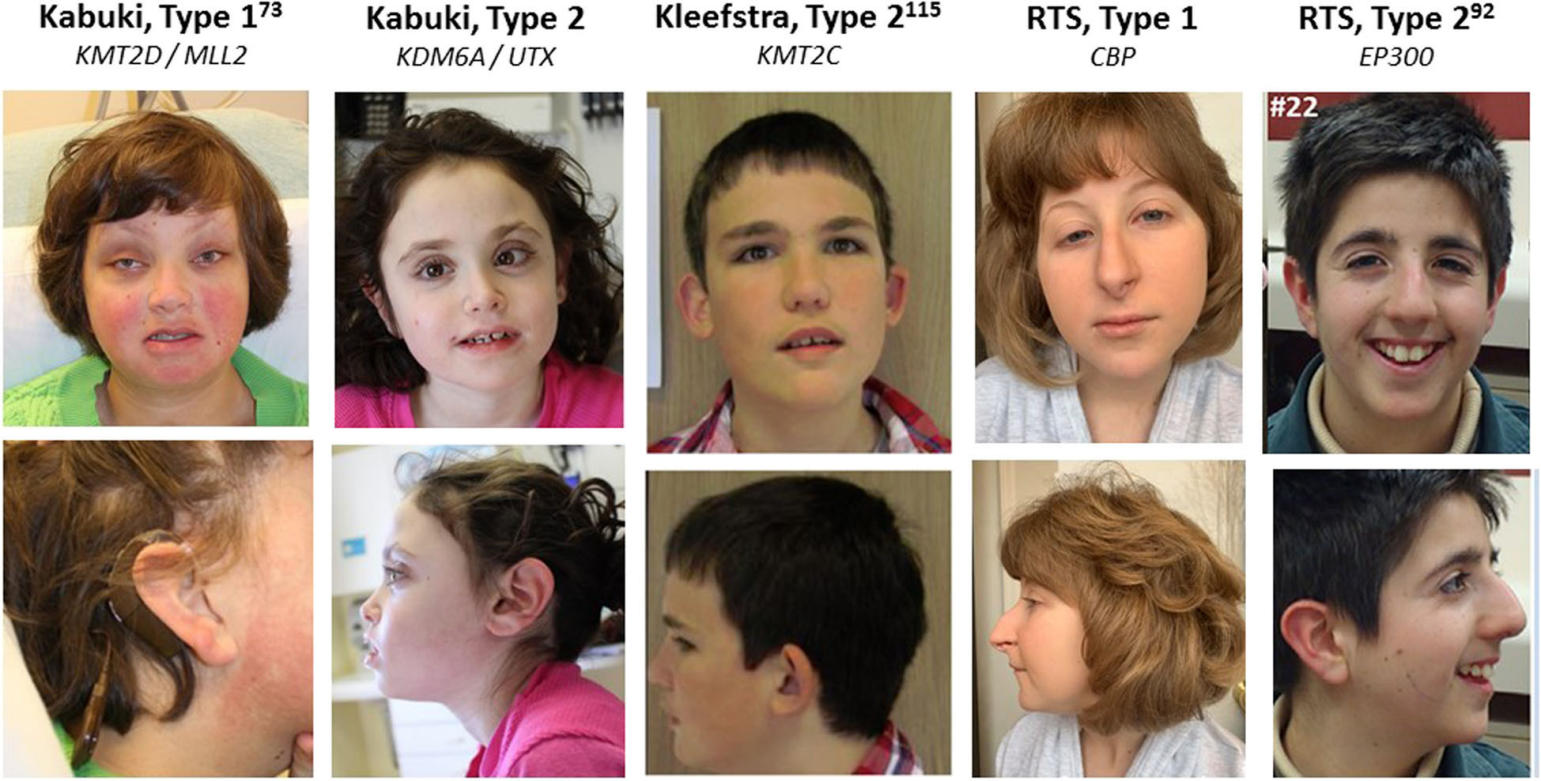
Craniofacial phenotype of K_CD_COM-associated diseases. Representative facial and profile photographs of patients with indicated diseases and mutations

Abnormal craniofacial development is a highly prevalent feature of all five K_CD_COM-associated diseases (Fig. 2). While some syndrome-specific facial features (KS, high arch eye-brows; RTS, prominent nasal septum extending below nares) are classically described in the literature, in clinical practice there is substantial overlap in the facial presentations observed across subjects diagnosed with these syndromes. No single specific dysmorphic feature is considered pathognomonic for any of these diagnoses and the penetrance/expressivity of individual characteristics is heterogeneous. Nevertheless, disruption of normal craniofacial development in general is a consistent finding across K_CD_COM-associated diseases, suggesting dysregulation of shared developmental pathways/morphogenic processes.

Embryonic development of the human face/anterior skull is a tightly regulated three-dimensional morphogenetic process involving the integrated fusion of five facial primordia (frontonasal prominence [FNP] plus bilaterally paired maxillary [MaxP] and mandibular [ManP] processes )[126]. These facial primordia develop following migration of dienecephalic- and mesencephalic-derived cranial neural crest cells (CNCCs) into the developing head (which generates the FNP), and first pharyngeal arch (which generate the MaxP, ManP), respectively. CNNC-derived tissues orchestrate the development of sensory ganglia and anterior facial bones and cartilage, including the periorbital structures, the nose, palate, and inner/middle/outer ear [127].

**Fig. 3:**
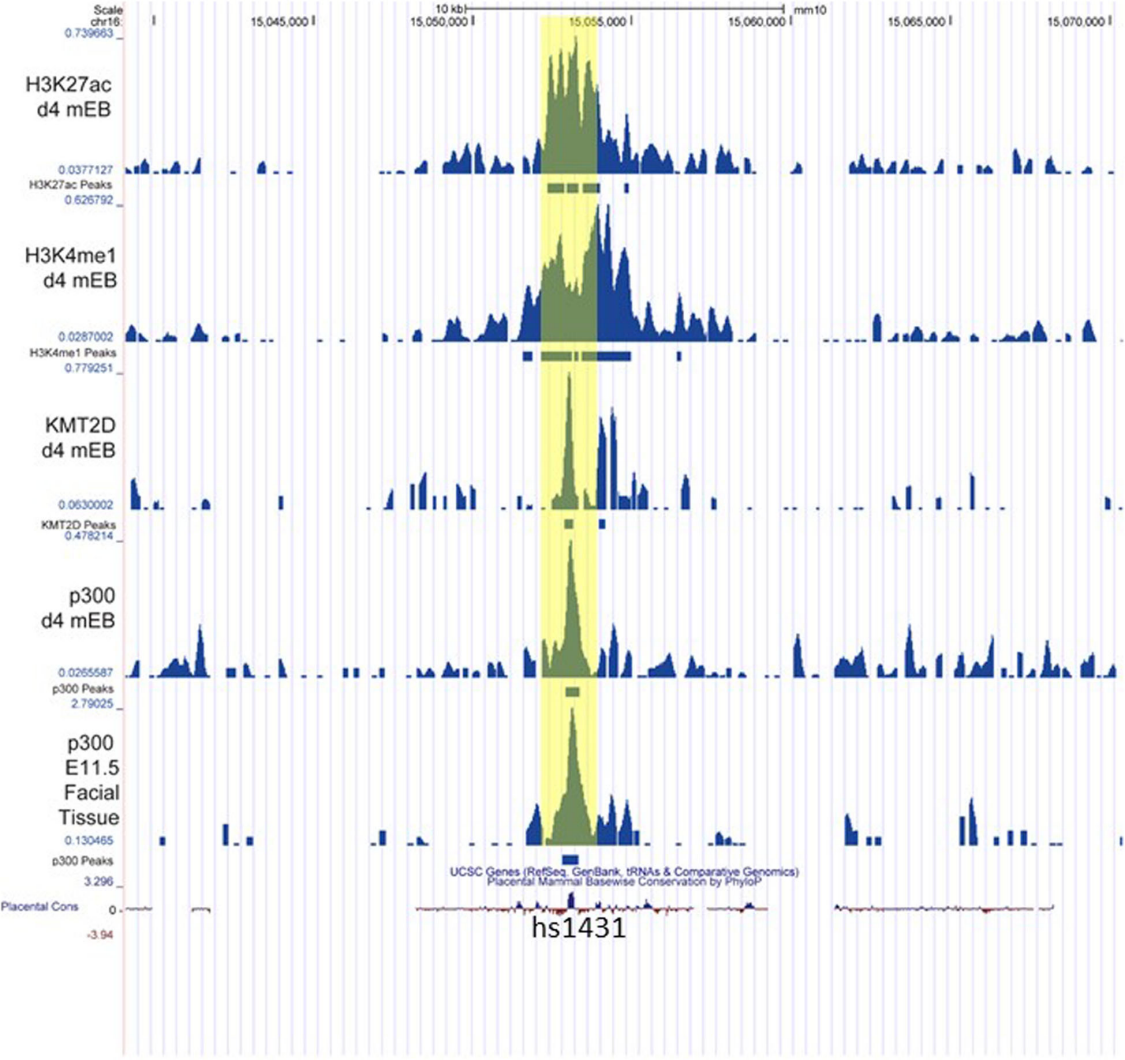
Epigenetic craniofacial enhancer analysis. Epigenetic analysis of conserved craniofacial enhancer element hs1431 shown as Genome Browser tracks of aligned indicated ChIP Seq signal peaks. Y-axis indicates coverage by estimated fragments normalized to millions of reads mapped generated from next generation sequencing. Experiments are from mouse 4 days old embryoid bodies (MLL3^−/−^;MLL4^flox/flox^) [48] and e11.5 embryonic mouse facial tissu e[128]. Data analyzed are publically available from Gene Expression Omnibus database (accession numbers GSE50534 and GSE49413). Data analysis, peak calling, and visualization were performed in BioWardrobe [130] with GRCm38/mm10 genome build

Epigenetic regulation of craniofacial development has been profiled in both murine and human embryonic tissues, identifying thousands of conserved putative regulatory regions [128, 129]. Transgenic *lacZ* reporter and enhancer element knockout-mice have validated the craniofacial expression patterns and in vivo activity of multiple enhancer elements discovered in this fashion. To build on these findings, we aligned putative p300+ enhancer sites from the FACEBASE dataset with KMT2D ChIP-seq data from 4-day-old mouse embryoid bodies (Fig. 3) [48, 128]. Strikingly, we found strong co-localization of a KMT2D+ and p300 peaks with the putative craniofacial enhancer element hs1431. From e11.5 onward, the hs1431 element drives robust lacZ reporter gene expression in all five facial primordia plus the developing ear. Knock-out of the hs1431 element resulted in significant reduction in expression of the downstream *Snai2*/*Slug* gene in nose and maxillary process of e13.5 mouse embryos [128]. SNAI2 is a zinc-finger transcriptional repressor which regulated CNCC migration and mutations in this gene are linked to Waardenburg syndrome type 2D (OMIM 608890) [131]. Intriguingly, embryonic RNA in situ hybridization studies also localize *Kmt2d* expression to calvarial osteoblasts, further implicating the gene with the pathophysiology of craniosynostosis, a common finding in Kabuki syndrome patient s[132].

In addition, mouse haploinsufficiency models of KS type 1 and RTS recapitulate the abnormal craniofacial development observed in patients with these diagnoses [85, 133]. In contrast, the mouse model of X-linked KS type-2 showed no craniofacial defects in the female UTX^+/−^ animals [134]. Lineage-specific experiments showed that conditional deletion of UTX in CNCC (using Wnt1-Cre) showed mild frontonasal hypoplasia in male animals (Utx^flox/y^) while severe craniofacial defects (including cleft palate) was observed in female homozygous (Utx^flox/flox^) animals. These results suggest that CNCCs may be particularly sensitive to UTX gene dosage and that the demethylase-dead UTY is able to partially compensate for loss of UTX [134].

A recent study of the skeletal phenotype in a KS-1 mouse model indicated that *Kmt2d* haploinsufficient mice have precocious chondrocyte differentiation and disrupted skeletal formation [135]. The mice have bowing of the ventral aspect of the skull and shortened long bones compared to wild-type controls, findings which recapitulate aspects of the human disease phenotype. The study demonstrated that there is increased expression of a *SOX9*, which is known to induce chondrogenesis in *Kmt2d*-deficient chondrocytes. This effect is likely mediated by putative KMT2D target *Shox2*, which releases *Sox9* inhibition. This study lends credence to the broader hypothesis that the various features of the KS phenotype are a direct mechanistic result of tissue-specific disruption of key tissue-specific developmental pathways that involve direct targeting of KMT2D or KDM6A. Further research in various tissues is necessary to elucidate these tissue-specific pathways.

## Emerging treatment strategies for K_CD_COM-ADs

The net functional effect of K_CD_COM activity is the activation or enhanced expression of targeted genes via (1) deposition of activating histone modification (H3K4m1, H3K4me3, and/or H3K27ac) on regulatory regions and/or (2) by acting as a scaffold for the assembly of transcription-promoting enhanceosomes. Thus far, proposed treatments strategies for K_CD_COM-ADs have primarily focused on the use of histone deacetylase inhibitors (HDACi’s), both to directly increase cellular H3K27ac levels as well as indirectly enhance H3K4me3 [136]. The HDACi suberoylanilide hydroxamic acid (vorinostat) was shown to reverse a defect in fear conditioning defect seen in a murine model of type 1 RTS [137]. Similarly, the HDACi AR-42 increased splenocyte H3K4me3 levels and improved spatial memory performance in in a type 1 KS mouse model [85]. In contrast to HDACi-based strategies, one study demonstrated that certain KMT2D nonsense mutations are responsive to gentamicin-induced “read through,” thus enhancing the expression of KMT2D target genes [138]. In addition to these approaches, new classes of histone-targeting drug compounds, including histone demethylase inhibitors [139], are currently being developed which may prove useful in the reducing the sequela of K_CD_COM-ADs.

Additional mechanistic insight into K_CD_COM-associated diseases might be gained by studying perturbations in disease phenotypes from pharmacologic interventions. For example, cellular mechanisms that support long term memory and cognition are well established to be mediated by CBP/EP300 signalin g[136]. cAMP is an activator of Protein Kinase A (PKA), which in turn is responsible for CREB phosphorylation. Phosphorylated CREB activates signaling pathways to potentiate memory formation and synaptic plasticity. Phosphodiesterase (PDE) inhibitors may potentially serve as treatments for cognitive dysfunction seen in K_CD_COM-associated diseases since they will reduce the rate of cAMP hydrolysis and thus increase intracellular cAMP levels, which would serve to increase PKA-mediated CREB signaling pathways [137]. This example illustrates the broader idea that pharmacologic intervention may help dissect mechanisms of K_CD_COM-associated diseases.

## Conclusion

A forty-two year gap separated the first clinical description of RTS and the identification of the two genes now known to drive ~ 50% of clinical cases. The linkage of CBP and EP300 to RTS was the product of incremental scientific advancement reflecting the technology of the day. Beginning with genetic analyses using karyotyping and fluorescent in situ hybridization (FISH) and ending with laborious gel-based Sanger sequencing, CBP haploinsufficiency was discovered in multiple RTS patients. This finding then logically suggested the potential involvement of CBP’s sister gene EP300. Ten years later, the first cases of EP300-linked RTS-2 were identified via new techniques (multiplex ligation-dependent probe amplification, MLPA) and improved sequencing technology (capillary-electrophoresis automated Sanger sequencing). KLEFS1’s association with EHMT1 mutations was revealed using a similar approach. In contrast, KS-1’s linkage to mutations in KMT2D (29 years after the syndrome was first described) would require the power of next generation sequencing of whole exomes. Surprisingly, even the brute force power of exomic sequencing, however, was incapable of detecting the Xp11.3 microdeletions disrupting KDM6A (KS-2), which were discovered via comparative genomic hybridization (CGH). With clinical whole genome sequencing now becoming increasingly available, the possibility of finding new causative mutations in regulatory regions has become a reality and along with it the daunting task of interpreting and validating pathologic variants identified in such large datasets.

Much as the discovery of the K_CD_COM-AD genes required the tireless application of diverse genetic tools for decades, the development of new treatments for these diseases will also not be simple or one-dimensional. While management of structural birth defects will likely remain the prevue of surgeons for the foreseeable future, many functional pathologies which affect K_CD_COM-AD patients may be feasible targets for pharmacologic therapy. Drugs targeting gene dysregulation in the central nervous system, the immune system, and post-natal stem cells may provide meaningful benefits for these patients. The talents and efforts of clinicians, molecular biologists, and pharmaceutical biochemists will be required to develop novel treatment strategies targeting K_CD_COM-AD functional pathology.

## Data Availability

Not applicable.

## Abbreviations

AE: Active enhancer
ASC, ASCOM: see K_CD_COM
ASH2L: Ash2 (trithorax-like) gene
ASHL2: absent, small, or homeotic 2-like
CBP or CREBBP: Creb binding protein
ChIP-Seq: Chromatin immunoprecipitation sequencing
CNCC: Cranial neural crest cells
CNS: Central nervous system
COMPASS: Complex of proteins associating with Set1
DPY-30: Dumpy-30
EHMT1: H3K9 methyltransferase, also known as KMT1D
EP300: Enzyme p300
FSIQ: Full scale intelligence quotient
H3K4me1/2/3: Histone 3 lysine 4 mono/di/tri methylation HAT Histone acetyl transferase
H3R2me2: Histone 2 arginine 2 dimethylation
HDACi: Histone deacetylase inhibitors
Hox: Homeobox
IQ: Intelligence quotient
K_CD_COM-AD: KMT2C/D COMPASS complex-associated diseases
KIS: Kabuki interaction surface
KIX: Kinase-inducible domain interacting domain
KMT2C/D: Type 2 lysine methyl transferase C/D
KS: Kabuki syndrome
KSS: Kleefstra syndrome
mESC: Mouse embryonic stem cell
MLL 3/4: See KMT2C/D
NCOA6: Nuclear receptor coactivator 6
NR: Nuclear receptor
OMIM: Online Mendelian Inheritance in Man
PAGR1/PA1: PAX-interacting protein1-associated glutamate rich protein 1a
PHD: Histone-binding plant homeodomain
PTIP: PAX interacting protein 1
RBBP5: Retinoblastoma binding protein 5
RNAi: RNA interference
RNA-Seq: RNA sequencing
RTS: Rubinstein-Taybi syndrome
SAM: S-adenosyl-l-methionine
SDI: Sdc1-Dpc-30 interaction
SET: Su(var)3-9, Enhancer-of-zeste, Trithorax
SPRY: spla and the ryanodine receptor
TSS: transcription start site
UTX: ubiquitously transcribed tetratricopeptide repeat, X chromosome
UTY: y-Chromosome paralog, KDM6C
WIM: Win interaction motif
WRAD: WDR5 (WD repeat domain 5)
